# Sentinel Lymph Node Detection in Early-Stage Oral Squamous Cell Carcinoma Using Magnetic Resonance Lymphography: A Pilot Study

**DOI:** 10.3390/jcm13237052

**Published:** 2024-11-22

**Authors:** Dominique N. V. Donders, Rutger Mahieu, Roosmarijn S. Tellman, Marielle E. P. Philippens, Robert J. J. van Es, Ellen M. Van Cann, Gerben E. Breimer, Remco de Bree, Bart de Keizer

**Affiliations:** 1 Department of Head and Neck Surgical Oncology, University Medical Center Utrecht, 3584 CX Utrecht, The Netherlands; d.n.v.donders@umcutrecht.nl (D.N.V.D.); r.mahieu@umcutrecht.nl (R.M.); r.s.tellman@umcutrecht.nl (R.S.T.); r.j.j.vanes@umcutrecht.nl (R.J.J.v.E.); e.m.vancann@umcutrecht.nl (E.M.V.C.); 2 Department of Radiotherapy, University Medical Center Utrecht, 3584 CX Utrecht, The Netherlands; m.philippens@umcutrecht.nl; 3 Department of Pathology, University Medical Center Utrecht, 3584 CX Utrecht, The Netherlands; g.e.breimer-2@umcutrecht.nl; 4 Department of Radiology and Nuclear Medicine, University Medical Center Utrecht, 3584 CX Utrecht, The Netherlands; b.dekeizer@umcutrecht.nl

**Keywords:** mouth neoplasms, sentinel lymph node biopsy, lymphatic metastasis, lymphography, magnetic resonance imaging

## Abstract

**Objectives:** To assess the efficacy of magnetic resonance (MR) lymphography with gadobutrol contrast for sentinel lymph node (SLN) mapping in early-stage oral squamous cell carcinoma (OSCC). **Methods:** This pilot study compared the identification of SLNs by MR lymphography using a gadolinium-based contrast agent (gadobutrol) to conventional [^99m^Tc]Tc-nanocolloid lymphoscintigraphy (including single-photon emission computed tomography/computed tomography (SPECT/CT)) in 10 early-stage OSCC patients undergoing SLN biopsy. The patients initially underwent conventional lymphoscintigraphy following the peritumoral administration of indocyanine green [^99m^Tc]Tc-nanocolloid (120 megabecquerel; ~0.5 mL). Subsequently, 0.5–1.0 mL gadobutrol was peritumorally injected, and MR imaging was acquired for 30 min. The following day, the identified SLNs were harvested and subjected to a histopathological assessment. The MR lymphography and [^99m^Tc]Tc-nanocolloid lymphoscintigraphy results were evaluated and compared with respect to those of the SLN identification. The reference standard consisted of a histopathological evaluation of the harvested SLNs, complementary neck dissection specimens, and follow-up data. **Results:** The MR lymphography detected 16 out of 27 SLNs identified by [^99m^Tc]Tc-nanocolloid lymphoscintigraphy, revealing an additional SLN that did not harbor metastasis. MR lymphography failed to identify any SLNs in one patient. Of the seven histopathologically positive SLNs detected by [^99m^Tc]Tc-nanocolloid lymphoscintigraphy, three were identified by MR lymphography. All patients remained disease-free after a median follow-up of 16 months. Compared to [^99m^Tc]Tc-nanocolloid lymphoscintigraphy, MR lymphography using gadobutrol achieved an SLN identification rate of 59%, a sensitivity of 75%, and a negative predictive value of 86%. **Conclusions:** MR lymphography using gadobutrol demonstrates limited reliability for SLN mapping in early-stage OSCC.

## 1. Introduction

A sentinel lymph node (SLN) biopsy is a well-established staging procedure for patients with clinically negative necks in early-stage oral squamous cell carcinoma (OSCC). Compared to elective neck dissection, SLN biopsy provides similar survival outcomes while significantly reducing functional morbidity [1,2,3]. Despite advances in SLN imaging techniques, such as single-photon emission computed tomography/computed tomography (SPECT/CT) [4], the utilization of conventional technetium-99m (^99m^Tc) labeled radiotracers in SLN biopsy still presents technical and logistical challenges. Moreover, it emits gamma radiation and is potentially harmful for both patients and healthcare workers. Therefore, ongoing investigations are being conducted to examine alternative high-resolution imaging techniques for SLN imaging [5,6,7].

A major challenge in SLN biopsy for OSCC is the “shine-through” phenomenon, in which radioactivity from the injection site overshadows nearby SLNs, particularly in tumors located in the floor of the mouth. This phenomenon reduces the sensitivity of SLN detection. Magnetic resonance (MR) lymphography, using gadolinium-based contrast agents, offers a high spatial resolution and a superior signal-to-noise ratio compared to conventional lymphoscintigraphy. This technique can potentially eliminate the shine-through effect by providing detailed anatomical images that allow for the reliable identification of SLNs located close to the injection site. Moreover, MR lymphography does not involve radiation exposure or require radioisotopes, making it a safer and more accessible option, especially in settings where nuclear medicine facilities are limited [8,9,10]. Recent studies have demonstrated the potential of interstitial contrast-enhanced magnetic resonance imaging (MRI) with extracellular gadolinium-based contrast agents for SLN mapping in patients with breast cancer and OSCC [11,12,13]. In a pioneering study involving OSCC patients, MR lymphography using a small volume (1 mL) of peritumorally administered gadobutrol consistently visualized SLNs in all 26 patients, with the majority (81%) also showing draining lymphatic vessels [11]. Among the 11 patients with pathologically positive necks, 10 were correctly staged using MR lymphography. However, in one case, MR lymphography identified SLNs in ipsilateral level III, while the final histopathology revealed metastases in ipsilateral level I without level III involvement.

Despite the promising findings of this study, significant concerns remain due to methodological limitations [11]. These include patient selection bias, the reliance on inferior reference standards, and the lack of follow-up data. For instance, SLNs identified by MR lymphography were ultrasound-guided injected with blue dye the day before surgery, and the blue stained SLNs were dissected from the elective neck dissection specimen one to two weeks after the MR lymphography. Additionally, the inclusion of larger tumors, which inherently have a higher risk of cervical metastases, may have skewed the results. Furthermore, the absence of follow-up data limits the assessment of the true accuracy of MR lymphography using gadolinium-based contrast agents. The histopathological examination in this study did not employ step serial sectioning or immunohistochemistry, increasing the risk of missing micrometastases [11].

The present study aims to provide a more rigorous comparison of the SLN identification rate and accuracy of MR lymphography using gadobutrol versus conventional lymphoscintigraphy in early-stage OSCC patients. By utilizing a detailed histopathological assessment and follow-up, particularly in cases with negative SLNB results, this study seeks to offer a clearer understanding of the true accuracy of MR lymphography with gadobutrol for SLN mapping.

## 2. Materials and Methods

### 2.1. Patients

This study was approved by the Ethics Committee of the University Medical Center Utrecht (no. 21/722) and is registered in the Netherlands Trial Register (NL9005, accessible via: https://www.trialregister.nl/, accessed on 15 October 2024).

A total of 10 patients were prospectively enrolled. The inclusion criteria required patients to have diagnosis of early-stage OSCC (cT1-2N0; TNM Staging Union for International Cancer Control (UICC) 8th Edition [14]). Selection was based on clinical and radiological valuations. Additionally, all patients were scheduled to undergo an SLN biopsy.

Clinical negative nodal staging was determined by palpation and ultrasound of the neck in all patients; ultrasound-guided fine-needle aspiration cytology (US-FNAC) was performed in those with enlarged and suspicious lymph nodes [15].

Exclusion criteria are patients with positive nodal staging, distant metastases, secondary cancers, or previous head and neck malignancies in the past five years. Additionally, patients with a history of significant neck injury that would hinder surgical dissection of SLNs, previous neck dissection, or radiotherapy to the neck were excluded. Furthermore, patients with a documented allergic reaction to gadolinium-based contrast agents, known claustrophobia, or severe renal impairment (eGFR < 30) were ineligible for enrollment in this study. 

### 2.2. Study Design

On the day before surgery, enrolled patients first received peritumoral injections of 120 megabecquerel (MBq) and 0.05 mg indocyanine green (ICG-)[^99m^Tc]Tc-nanocolloid in a volume of ~0.5 mL, followed by lymphoscintigraphy, including SPECT/CT. Following lymphoscintigraphy, the location of identified SLNs was marked on the overlying skin using a 57Co penpoint marker and handheld gamma camera (Crystal Cam, Crystal Photonics GmbH, Berlin, Germany) [16].

After conventional lymphoscintigraphy, a total of ~1.0 mL undiluted gadobutrol was injected peritumorally, which was immediately followed by MRI acquisition with a total duration of 30 min.

The following day, the marked SLNs were harvested under conventional gamma-probe (Europrobe 3; Eurorad S.A., Eckbolsheim, France) and fluorescence guidance (PDE NEO II near-infrared camera; Hamamatsu, Herrsching, Germany). Any additional SLNs identified by MR lymphography were harvested based on their anatomical location. The location of excised SLNs and their counts per second and ICG uptake, as measured by the portable gamma probe and PDE NEO II near-infrared camera, were registered.

Harvested SLNs underwent histopathological examination utilizing step serial sectioning (~150 μm section thickness) and staining with hematoxylin–eosin (HE), and, if no metastasis was identified on the HE slides, pan-cytokeratin antibody (AE 1/3) [17,18]. Histopathologically positive lymph nodes were classified according to Hermanek et al. [19]: isolated tumor cells (ITCs) if their diameter was less than 0.2 mm, micrometastases if the diameter was measured to be between 0.2 mm and 2.0 mm, and macrometastases if the metastatic tumor tissue exceeded 2.0 mm in diameter.

Patients with histopathologically negative SLN(s) were managed with a wait-and-scan approach during follow-up. For those with at least one histopathologically positive SLN, complementary treatment of the affected and adjacent nodal basins was applied (i.e., neck dissection). Complementary neck dissection specimens underwent histopathological assessment to detect additional (non-sentinel) nodal metastases.

### 2.3. Peritumoral Injection of Contrast Agents

In this study, patients received peritumoral injections of indocyanine green (ICG-) [^99m^Tc]Tc-nanocolloid with a volume of ~0.5 mL and undiluted gadolinium-based contrast agent (gadobutrol/Gadovist^TM^) with a volume of ~1.0 mL, both at room temperature.

Peritumoral injections with both tracers were administered by the same physician in an identical manner to ensure procedural consistency. The peritumoral injections were performed under direct visualization of the tumor, with 2 to 4 injections given depending on tumor size and accessibility. The injections were distributed across four quadrants surrounding the tumor, using a compatible 1 mL syringe and a 25 G needle.

### 2.4. MR Lymphography

MRI scanning was conducted using a 3 Tesla (3T) MRI scanner (Philips Healthcare, Best, the Netherlands), using a head receive coil and an anterior receive coil. MR lymphography consisted of water-only images of 3D dynamic multiple Dixon T1-weighted spoiled gradient echo (T1 mDixon SPGR) scan (TE: 1.52/3.0 ms, TR: 4.7 ms, flip angle: 10°, SENSE: 1.4, resolution: 1.2 × 1.2 × 2.0 mm^3^) for 10 min. This was followed by water-only images of 3D multiple Dixon T1-weighted spoiled gradient echo (T1 mDixon SPGR) scan (TE: 1.94/3.4 ms, TR: 5.6 ms, flip angle: 10°, SENSE: 1.1, resolution: 1 × 1 × 1 mm^3^), water-only images of coronal T1 turbo spin echo (c T1 TSE) scan (TE: 14/15 ms, TR: 584 ms, SENSE: 2, resolution: 0.9 × 1.0 mm^2^, slice thickness: 3 mm), and water-only images of multiple Dixon T2-weighted turbo spin echo (T2 mDixon TSE) scan (TE: 100/101 ms, TR: 3000 ms, SENSE: 2, resolution: 1.3 × 1.2 mm^2^, slice thickness: 3 mm). All MR sequences for anatomical imaging are correlated with MR lymphography.

### 2.5. Scintigraphy and SPECT/CT

Conventional lymphoscintigraphy consisted of early dynamic and static scintigraphy, followed by late static scintigraphy and SPECT/CT acquisition two hours post injection, according to European Association of Nuclear Medicine (EANM) guidelines [20].

Dynamic planar scintigraphic images were obtained with a 128 × 128 matrix: 60 frames of 30 s each in anterior/posterior projection, followed by static mode (256 × 256 matrix) for 4 min in both anterior/posterior and lateral projections. Static images were acquired at 30 min and 2 h post injection. Additionally, 30 s flood field images were acquired to supplement dynamic and static scintigraphic imaging.

SPECT/CT imaging was performed using a 128 × 128 matrix with a pixel spacing of 4.8 × 4.8 mm^2^, encompassing 128 angles with 20 s per projection, over a non-circular 360° orbit. CT parameters were set at 110 kV, 40 mAs effective dose, and a slice thickness of 1.2 mm.

Lymphoscintigraphy was conducted with a Symbia^TM^ T16 SPECT/CT scanner (Siemens Healthineers, Erlangen, Germany) with ‘low- and medium energy’ collimators, aimed at limiting septal penetration and minimizing shine-through artifacts [21]. SPECT/CT images were reconstructed using clinical reconstruction software (Siemens Flash3D, https://xcelerator.siemens.com/global/en/all-offerings/products/3/3d-flash.html), incorporating attenuation and scatter correction with parameters set at 6 iterations, 8 subsets, and a 5 mm Gaussian filter.

### 2.6. Evaluation and Analyses

Each patient’s MR lymphographic images and conventional [^99m^Tc]Tc-nanocolloid lymphoscintigraphy images were assessed to determine the similarity of the depicted draining lymph node basins, as well as the number and location of SLNs. Additionally, comparisons were made between MR lymphography and conventional lymphoscintigraphy regarding the visualization of draining lymphatic vessels transporting the (radio)tracer.

SLNs identified by either MR lymphography or conventional lymphoscintigraphy were correlated with findings from histopathological examination of excised SLNs and any complementary neck dissection specimens, as well as follow-up results, in particular, regional recurrences in absence of local recurrence. All images were reviewed by a radiologist/nuclear medicine physician (B.K.) and a second observer (D.N.V.D.).

The diagnostic accuracy of MR lymphography was assessed using histopathological examination of harvested SLNs and complementary neck dissection specimens, along with follow-up data as the reference standard. Sensitivity (true positives/(true positives + false negatives)) and negative predictive value (NPV) (true negatives/(true negatives + false negatives)) were calculated.

Adverse reactions were scored and graded according to the Common Terminology Criteria for Adverse Events (CTCAE) [22].

All data were analyzed using IBM SPSS Statistics Version 29.0.1. Categorical variables are presented as the number of cases and percentages. Continuous parametric variables are presented as mean (±standard deviation (SD)), while non-parametric variables are presented as median. Statistical tests were not performed due to the small number of patients in this study.

## 3. Results

Between April 2022 and June 2023, a total of 10 patients diagnosed with early-stage OSCC (cT1-2N0; TNM Staging UICC 8th Edition [14]) and scheduled for SLN biopsy were prospectively included in this study.

Additional diagnostic MRIs of the head and neck were performed on four out of the ten patients.

The patient and tumor characteristics of the included patients are summarized in Table 1. The majority of the patients presented with an OSCC of the oral tongue (60%). Most of the tumors were classified as cT1 (70%). A total of forty-one SLNs were harvested, with a median of four SLNs per patient, among which seven contained metastases (17%). Out of these seven SLNs, four histopathologically positive lymph nodes contained macrometastases, two contained micrometastases, and one contained ITC only.

These occult lymph node metastases were detected in four out of ten patients by an SLN biopsy. Among those with histopathologically positive SLNs, three underwent complementary neck dissection (75%), two of whom also underwent postoperative radiotherapy following complementary neck dissection, due to pN2 staging, and one patient refused postoperative radiotherapy. The remaining patient adopted a wait-and-scan approach due to ITC. A subsequent histopathological examination of complementary neck dissection specimens revealed no additional nodal metastases. All included patients remained disease-free after a median follow-up of 16 months (with a range of 7–21 months).

### Sentinel Lymph Node Identification on MR Lymphography

**Figure 1:**
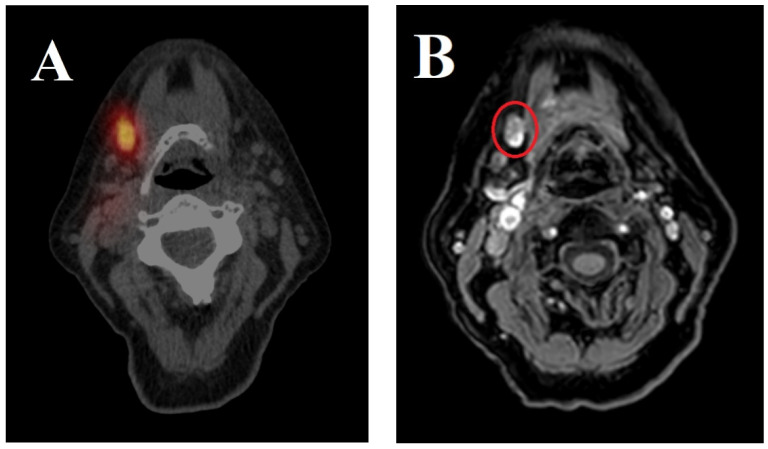
Conventional [^99m^Tc]Tc-nanocolloid lymphoscintigraphy and gadobutrol-enhanced MR lymphography. (**A**) Conventional [^99m^Tc]Tc-nanocolloid lymphoscintigraphy of a patient diagnosed with cT1N0 OSCC on the right side of the oral tongue (patient 2; Table 2), sentinel lymph node in level Ib on the right side with uptake of [^99m^Tc]Tc-nanocolloid. (**B**) Gadobutrol-enhanced MR lymphography for the same patient depicting the sentinel lymph node in level Ib on the right side (red circle).

The results of the MR lymphography and [^99m^Tc]Tc-nanocolloid lymphoscintigraphy for all enrolled patients are presented in Table 2 and an example is shown in Figure 1.

Most of the detected SLNs in the MR lymphography were found in level II (10 out of 17 detected SLNs). [^99m^Tc]Tc-nanocolloid lymphoscintigraphy detected a total of 27 SLNs, of which 16 SLNs (59%) were also identified by MR lymphography. Additionally, the MR lymphography identified one extra SLN (patient 8, level IIa), which was harvested intra-operatively based on its anatomical location. This SLN exhibited no histopathological evidence of metastasis. In one patient, MR lymphography failed to detect any SLNs (10%). Among the seven histopathologically positive SLNs detected by conventional lymphoscintigraphy in four patients, three SLNs were identified by MR lymphography (43%) in three patients.

Overall, the MR lymphography using gadobutrol reached a sensitivity of 75% (3/4) and an NPV of 85.7% (6/7) when a histopathological assessment of the harvested SLNs and a specimen from the complementary neck dissection, along with follow-up data, were used as reference standards.

**Figure 2:**
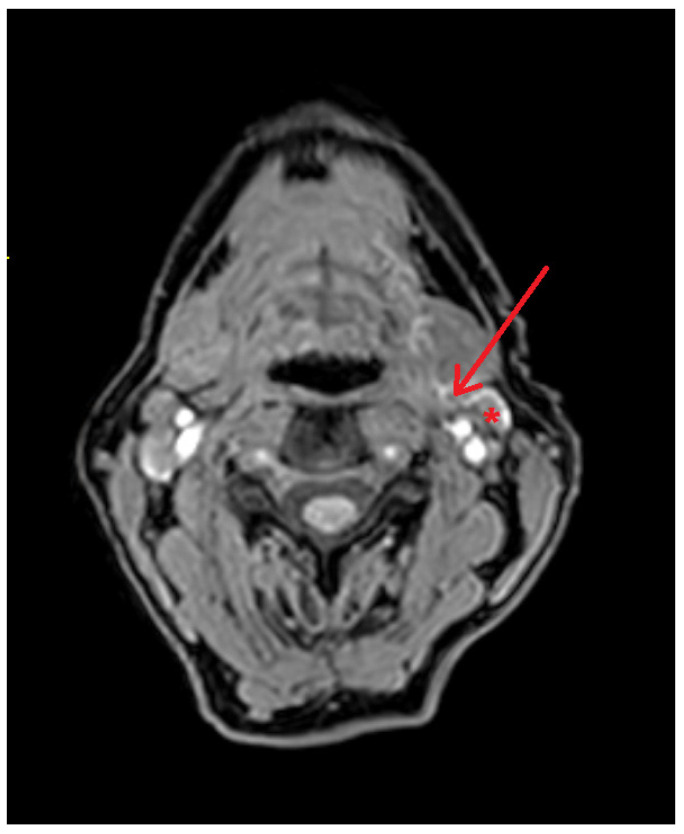
Gadobutrol-enhanced MR lymphography with lymphatic vessel drainage. Gadobutrol-enhanced MR lymphography of a patient diagnosed with cT1N0 OSCC on the left side of the oral tongue (patient 9; Table 2) depicting the sentinel lymph node in level IIa on the left side (*) and lymphatic drainage with uptake of gadobutrol (red arrow).

Contrast-enhanced lymphatic vessels were visualized in two patients with MR lymphography (20%), whereas conventional lymphoscintigraphy successfully visualized lymphatic vessel drainage of [^99m^Tc]Tc-nanocolloid in four patients (40%) (Figure 2).

**Figure 3:**
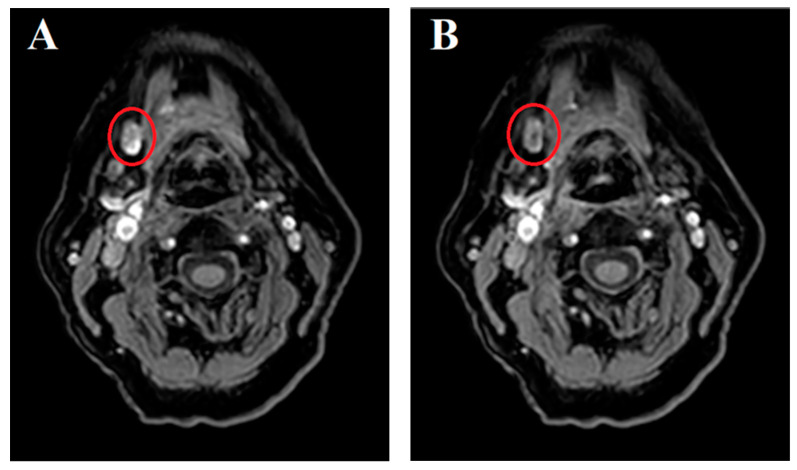
Dynamic MR lymphography. Contrast-enhanced dynamic MR images acquired for 10 min after peritumoral injection of undiluted gadobutrol in the same patient as Figure 1 (patient 2; Table 2). Rapid lymphatic drainage of gadobutrol was observed through dynamic MR lymphography, with gadobutrol washed out from the SLN within 7 min. (**A**) Initial dynamic MR depicting the sentinel lymph node in level Ib on the right side (red circle). (**B**) MR imaging acquired at 6 min and 23 s following (**A**), showing the same SLN in level Ib on the right side (red circle).

Upon dynamic imaging, brief SLN contrast enhancement with fast tracer washout was observed in five of the seventeen detected SLNs by MR lymphography, as depicted in Figure 3.

In one patient with a tumor located in the retromolar trigone, an injection volume of ~1.0 mL was not feasible, and ~0.5 mL gadobutrol was injected. Mild swelling and some discomfort from the peritumoral injection with gadobutrol was observed in eight patients, corresponding with CTCAE grade 1. No severe adverse reactions were reported following the peritumoral injections of gadobutrol or MR lymphography.

**Figure 4:**
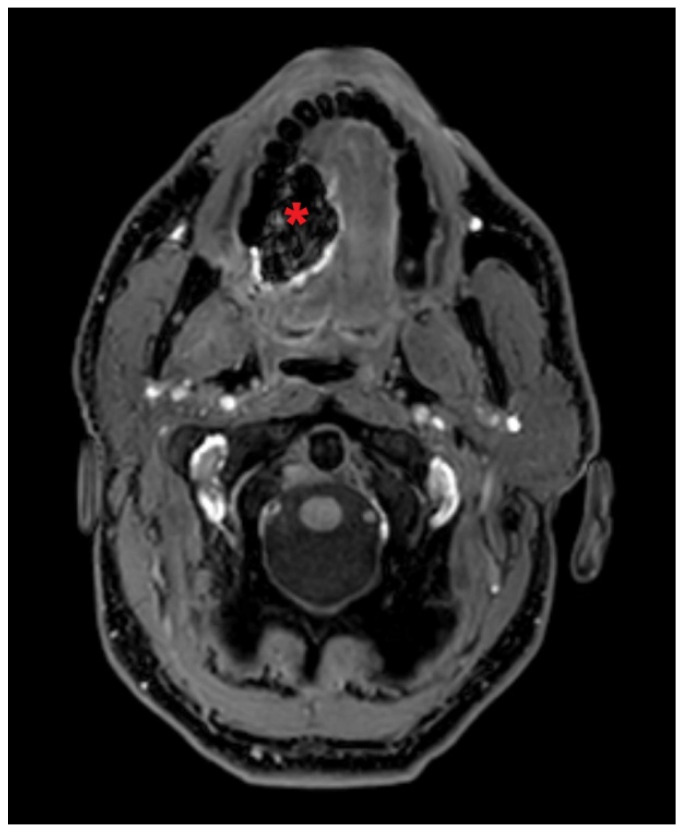
MR lymphography with paramagnetic artifact due to [^99m^Tc]Tc-nanocolloid. Gadobutrol-enhanced MR lymphography in a patient diagnosed with cT1N0 OSCC on the right side of the oral tongue (patient 4), showing an artifact of [^99m^Tc]Tc-nanocolloid radiotracer at the injection site (*).

In all patients, paramagnetic artifacts from previous [^99m^Tc]Tc-nanocolloid injections were observed in proximity to the injection site (Figure 4).

## 4. Discussion

In this pilot study, we evaluated the efficacy of magnetic resonance (MR) lymphography using a gadolinium-based contrast agent for sentinel lymph node (SLN) mapping in patients with early-stage oral squamous cell carcinoma (OSCC). Our findings revealed that MR lymphography achieved a relatively low SLN identification rate of only 59%, and it failed to detect four out of seven histopathologically positive SLNs (57%). Notably, in one patient (10%), no contrast-enhanced SLN was detected, and in another, both histopathologically positive SLNs were missed, which would have led to false-negative staging if MR lymphography had been the sole diagnostic modality. The low sensitivity observed in this study, failing to detect 57% of histopathologically positive SLNs, strongly suggests that MR lymphography is unsuitable as a standalone SLNB method.

These results are in contrast to those reported by Bae et al., who achieved a higher SLN detection rate and a 91% success rate in identifying patients with pathologically positive necks using gadolinium-enhanced MR lymphography [11]. The discrepancies between our findings and those of Bae et al. may be attributed to several key differences in study design and methodology. Bae et al. included larger tumors (with a median size of 2.2 cm, ranging from 1.0 to 4.6 cm), specifically cT3 and cT4 OSCC, which inherently have a higher likelihood of cervical metastases. This difference in tumor size and stage could introduce a selection bias that may have inflated the detection rates reported in their study. Additionally, Bae et al. relied on elective neck dissection as the reference standard, which, while effective, does not utilize step serial sectioning or immunohistochemistry—techniques that significantly improve the detection accuracy of micrometastases and reduce the risk of false negatives. Our study employed a more rigorous reference standard, utilizing step serial sectioning and immunohistochemistry for a detailed histopathological assessment. This method is known to improve the detection of micrometastases and upstage the nodal status in up to 15.2–19.5% of patients [23,24]. Additionally, the study by Bae et al. also does not provide follow-up results, resulting in the inability to identify false-negative cases over time. This suggests that the reported successes in other studies may not be reproducible in broader clinical practice, reducing the utility of continued research in this area.

Several drawbacks of MR lymphography with gadobutrol were encountered in this study. In particular, the rapid lymphatic transportation of gadobutrol was observed with minimal retention in SLNs and rapid tracer washout (Figure 3). This is considered to be due to the low molecular weight of gadolinium-based contrast agents [25,26] and could increase the risk of overlooking SLNs and erroneously designating contrast-enhanced higher-echelon nodes as SLNs [27]. Rapid drainage was observed even when gadobutrol was administered without dilution, as in our study, which differs from the methodology employed by Bae et al. [11]. We anticipated that drainage would be slower with the undiluted contrast agent compared to their diluted mixture, yet even with the undiluted gadobutrol, the lymphatic drainage may be too fast to allow for reliable SLN identification.

In addition, it must be emphasized that there is currently no method to detect gadolinium-based contrast agents during surgery. Without reliable tools for intraoperative localization, MR lymphography risks misidentifying SLNs, potentially leading to suboptimal surgical outcomes. As a consequence, MR lymphography using gadobutrol is dependent on less reliable dual-tracer methods [28]. The challenge of detecting SLNs by MR lymphography and localizing them intraoperatively may be solved by using superparamagnetic iron oxide (SPIO). SPIO can be detected by both MRI and a handheld magnetometer, offering a potential method for the intraoperative localization of SLNs [29,30]. The initial results of MR lymphography using SPIO are promising, as all SLNs identified by MR lymphography corresponded with those identified by conventional lymphoscintigraphy [30,31]. Currently, the limited number of early-stage OSCC patients who have undergone MR lymphography with SPIO in our center prevents the assessment of its diagnostic accuracy. Larger studies with adequate reference standards (i.e., a combination of a detailed histopathological assessment and follow-up) should be conducted.

Another drawback of MR lymphography using gadobutrol is the swelling, pain, and discomfort following the peritumoral injection of gadobutrol. This reaction is likely attributable to the lower osmolarity of gadobutrol compared to [^99mTc^]Tc-nanocolloid [32]. Osmolarity plays a significant role in determining the tolerability of contrast agents, with low-osmolar agents generally associated with a reduced risk of adverse reactions [15,33,34]. The injection pain and swelling associated with the administration of gaobutrol, despite being manageable, represent significant drawbacks compared to other SLNB methods that deploy more tolerable agents.

Still, if the current shortcomings of MR lymphography can be overcome, it could further benefit oncological practice. For instance, in patients in whom MRI of the head and neck has already been acquired as part of clinical staging, the addition of a viable peritumorally injected MR contrast agent could facilitate the selection of lymph nodes with the highest risk of (occult) metastases (i.e., SLNs) for US-FNAC, thus enhancing the accuracy of US-FNAC and improving the selection of patients for either SLN biopsy in case of cN0 or a therapeutic neck dissection in the case of cN+. Similarly, since MRI is already increasingly used for radiotherapy planning [7], MR lymphography could be used to adapt radiotherapy planning, with a higher radiation dose targeted to lymph nodes (areas) with the highest risk of harboring occult metastases [35,36,37,38]. These potential applications underscore the importance of further research into MR lymphography, as it promises to enhance diagnostic and therapeutic approaches in early-stage OSCC.

There are several limitations of this study. First, due to the small number of included patients in this pilot study, our results should be interpreted with caution. Moreover, it should be realized that the results of this study cannot be fully compared to those achieved by Bae et al. on account of some discrepancies in methods [11]. Furthermore, to ensure that the standard of care for our patients was not compromised, MR lymphography was conducted after conventional lymphoscintigraphy. As a result, artifacts were observed by MR lymphography, presumably induced by [^99mTc^]Tc-nanocolloid (Figure 4). Artifacts related to [^99mTc^]Tc-nanocolloid on MRI scans have not been previously documented in the literature, but may have hindered the detection of SLNs by MR lymphography in this study. These artifacts undermine the reliability of the technique and suggest that its complexity and dependence on multiple tracers make it less practical for routine use compared to more straightforward methods.

Noteworthy, the average number of harvested SLNs in this study was relatively high [39]. This could be attributed to the supplementation of ICG to [^99mTc^]Tc-nanocolloid and the use of intraoperative near-infrared imaging. As previously demonstrated by Berger et al. [40], the use of intraoperative near-infrared imaging in melanomas of the head and neck region resulted in an increased number of harvested lymph nodes. Yet, in the study of the cohort of Berger et al., in which ICG was available, no significant decline in false-negative cases was seen, irrespective of the higher yield, raising questions about whether these additionally harvested lymph nodes based on a fluorescent signal were true SLNs. During surgery, many lymph nodes turn out to be fluorescent, sometimes without the presence of radioactive deposition. Consequently, in our study, surgeons may have excised more lymph nodes based on this visual signal. The relatively high number of harvested SLNs based on the conventional procedure in this study may negatively affect the SLN identification rate by MR lymphography. If many lymph nodes are excised and considered as SLNs, the SLN detection level of MR lymphography (relative to harvested SLNs) naturally shifts in disfavor of MR lymphography.. 

The strengths of our study include the homogeneous early-stage OSCC patient group, the within-patient comparison with conventional lymphoscintigraphy, and the use of an adequate reference standard (i.e., a detailed histopathological assessment after SLN biopsy, complementary treatment, and follow-up data).

## 5. Conclusions

In its current form, MR lymphography with gadobutrol presents significant limitations, including low sensitivity, poor intraoperative feasibility, rapid tracer washout, injection-related discomfort, and susceptibility to imaging artifacts. These shortcomings highlight the impracticality of this approach compared to well-established SLNB methods. Given these considerable barriers, further research into gadobutrol-enhanced MR lymphography appears unlikely to result in meaningful clinical improvements, and resources might be better allocated to refining existing techniques or exploring more promising alternatives. 

## Figures and Tables

**Table 1:** Patient and tumor characteristics.

Characteristics	*n* = 10
Gender, *n* (%)	
Female	6 (60%)
Median age (years) (range)	62.3 (51–80)
Tumor location, *n* (%)	
Tongue	6 (60%)
Floor of mouth	2 (20%)
Buccal mucosa	1 (10%)
Retromolar trigone	1 (10%)
Side primary tumor, *n* (%)	
Left	4 (40%)
Right	6 (60%)
Clinical T-stage, *n* (%) *	
T1	7 (70%)
T2	3 (30%)
Pathology primary tumor	
Mean diameter (mm) (SD)	14.7 (±7.4)
Mean depth of invasion (mm) (SD)	4.1 (±2.2)
Median harvested SLNs (range)	4 (1–7)
Histopathological status SLNs, *n* (%)	
Negative	34 (82.9%)
Positive	7 (17.1%)
Pathological N-stage after SLN biopsy, *n* (%) *	
pN0(sn)	6 (60%)
pN1(sn)	1 (10%)
pN2b(sn)	2 (20%)
pN2c(sn)	1 (10%)
Complementary neck treatment, *n* (%)	
Complementary neck dissection	3 (30%)
Complementary radiotherapy	2 (20%)
Follow-up (months) (range)	16.0 (7–21)

*n*, number; mm, millimeters; SD, standard deviation; SLNs, sentinel lymph nodes. * According to AJCC TNM classification, 8th edition.

**Table 2:** Comparison of sentinel lymph node distribution between CT lymphography and ^99m^Tc-nanocolloid lymphoscintigraphy.

N°	Primary Tumor	Identified SLNs LSG and SPECT/CT		Identified SLNs MR Lymphography		Harvested SLNs (cps)		PA	Complementary Treatment of Neck	pTNM Stage *	Upstaging Due to MR Lymphography	Follow-Up
	(Side)	Level	Side	Level	Side	Level	Side (cps)					(months)
1	Tongue (left)	Ib	Right			Ib	Right (192)	-	None	pT1N0(i+)(sn) cM0	No	NED [21]
		II/III	Right	II	Right	IIa	Right (426)	-				
		Ib	Left	Ib	Left	Ib	Left (1265)	+ ITC				
						Ia	Left (115)	-				
		II	Left			II/III	Left (118)	-				
						IIa	Left (133)	-				
		IV	Left	IV	Left	IV	Left (1458)	-				
2	Tongue (right)	Ib	Right	Ib	Right	Ib	Right (1834)	-	N.A.	pT1N0(sn) cM0	No	NED [20]
						Ib	Right (1100)	-				
		II	Right	II	Right	II	Right (6700)	-				
						II	Right (2331)	-				
3	Floor of mouth (left)	Ib	Right	Ib	Right	Ib	Right (340)	+macro	SND I–IV bilateral, RT	pT1N2c cM0	No	NED [17]
		Ib	Left			Ib	Left (857)	+macro				
		IV	Left			IV	Left (421)	-				
4	Tongue (right)	IIa	Right	IIa	Right	II	Right (1694)	-	N.A.	pT1N0(sn) cM0	No	NED [20]
		IIb	Right	IIb	Right	II	Right (6700)	-				
						II	Right (1162)	-				
		III	Right	III	Right	III	Right (3200)	-				
5	Buccal mucosa (right)	Ib	Right			Ib	Right (74)	-	N.A.	pT2N0(sn) cM0	No	NED [18]
		Ib	Right			Ib	Right (485)	-				
						Ib	Right (605)	-				
		II	Right			IIa	Right (398)	-				
6	Retromolar trigone (right)	Ib	Right	Ib	Right	Ib	Right (55)	-	MRND right	pT2N2b(mi) cM0	No	NED [17]
		IIa	Left			II/III	Right (129)	+micro				
						II/III	Right (88)	+micro				
7	Tongue (left)	II	Left	II	Left	II	Left (1050)	-	N.A.	pT2N0(sn) cM0	No	NED [16]
						II	Left (300)	-				
		III/IV	Left	III/IV	Left	IV	Left (9200)	-				
8	Floor of mouth (right)	Ib	Right			Ib	Right (20)	-	N.A.	pT1N0(sn) cM0	No	NED [15]
		II	Right	II	Right	IIa	Right (799)	-				
				II	Right	IIa	Right (729)	-				
						IIa	Right (63)	-				
		II	Left			IIa	Left (274)	-				
9	Tongue (left)	IIa	Left	IIa		II	Left (3740)	-	N.A.	pT1N0(sn) cM0	No	NED [9]
10	Tongue (right)	IIa	Right	IIa	Right	IIa	Right (1683)	+macro	SND I–IV right, RT	pT1N2b cM0	No	NED [7]
		IIa	Right			IIa	Right (1800)	-				
						IIa	Right (2168)	+macro				
						IIa	Right (242)	-				
						IIa	Right (443)	-				
		IIb	Right	IIb	Right	IIb	Right (1520)	-				
						III	Right (338)	-				

N°; patient number; SLN, sentinel lymph node; SPECT/CT, single-photon emission computed tomography/computed tomography; cps, counts per second as measured by conventional gamma probe; PA, pathological assessment; +, histopathologically positive for metastasis; -, histopathologically negative for metastasis; RT, radiotherapy; N.A., not applicable; MRND, modified radical neck dissection; SND, selective neck dissection. * According to AJCC TNM classification, 8th edition.

## Data Availability

The data presented in this study are available on request from the corresponding author.
